# Are kinocilia motile?

**DOI:** 10.7554/eLife.111609

**Published:** 2026-05-05

**Authors:** Ruth Anne Eatock, Marina Kabirova

**Affiliations:** 1 Department of Neurobiology, University of Chicago Chicago United States

**Keywords:** hair cell, kinocilia, motile cilia, vestibular inner ear, axoneme, bullfrog, Human, Mouse, Other

## Abstract

Gene expression patterns in the inner ear put an old question about structures called kinocilia back in motion.

**Related research article** Xu Z, Tavakoli A, Kulasooriya S, Liu H, Tu S, Bloom C, Li Y, Johnson TD, Zuo J, Tao L, Kachar B, He DZ. 2026. The dual molecular identity of vestibular kinocilia bridges structural and functional traits of primary and motile cilia. *eLife* **14**:RP108071. doi: 10.7554/eLife.108071.

Bundles of tiny hair-like structures have crucial roles in the inner ear. These hair bundles protrude from the top surfaces of sensory cells called hair cells, and convert motion into electrical signals that are sent to the brain (a process known as transduction). None of the structures within a hair bundle are actually hairs. Most are specialized microvilli, tightly linked to each other and aligned in rows behind a distinct element called the kinocilium (Figure 1A). The microvilli contain the proteins that detect the bundle motion caused by their specific stimuli: sound for auditory hair cells, and motions of the head for vestibular hair cells. The microvilli are also filled with filaments of actin that are cross-linked to make them very stiff.

**Figure 1.**
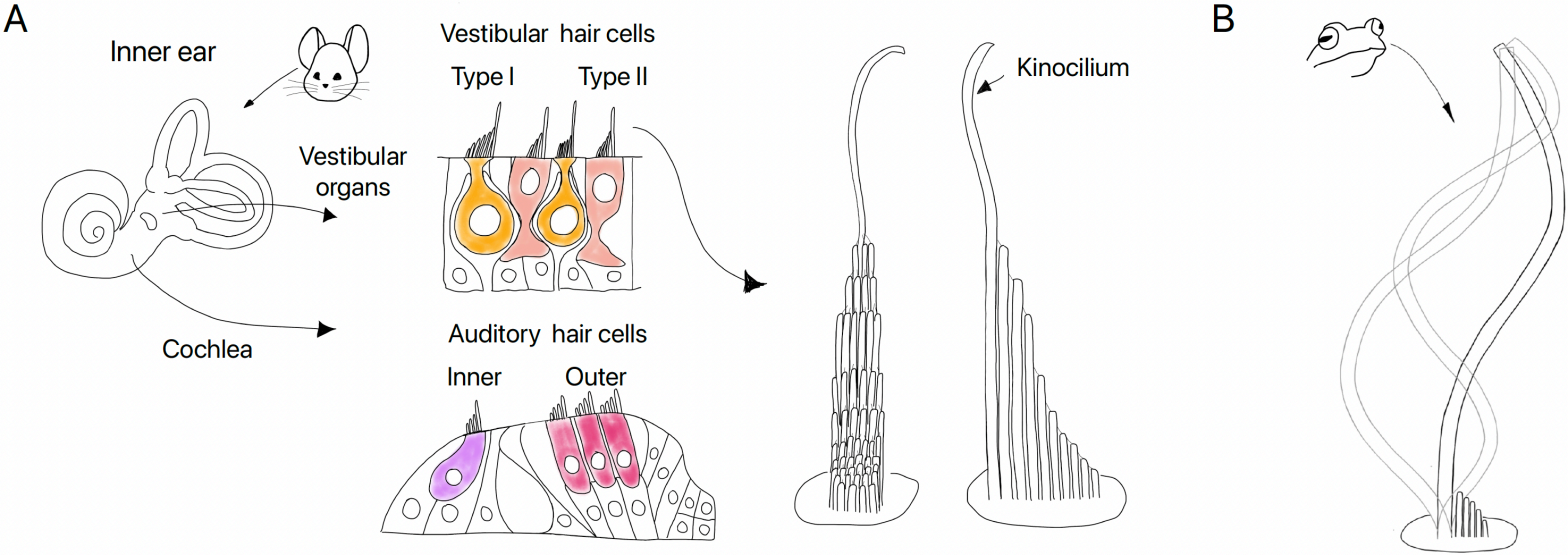
The kinocilia of vestibular hair cells can show motility, but the effects of this motility on hair cell function remain unknown. (**A**) Xu et al. studied gene expression in four types of hair cell (center panel) isolated from the inner ear organs (left) of mature mice: type I (orange) and type II (pink) hair cells from the vestibular organs, and inner (purple) and outer (red) auditory hair cells from the cochlea. The top surface of each vestibular hair cell is covered with rows of microvilli that rise in height to the single kinocilium (right; the bundle is shown from behind and the side). Although mature auditory hair cells do not have kinocilia, they retain the internal basal bodies of the kinocilia that were present in immature auditory hair cells. All hair cells are excited by deflection of the bundle toward its tall edge. (**B**) Xu et al. isolated vestibular hair cells from a bull frog, and observed the kinocilia on some cells performing a whip-like motion characteristic of motile cilia.

Although microvilli are usually referred to as ‘stereocilia’ in the literature, they are not cilia because they do not contain structures called axonemes (highly organized arrays of microtubules) at their core. The kinocilium is the only element in the hair bundle that is a true cilium, and it is not implicated directly in mature hair cell transduction (Hudspeth and Jacobs, 1979). However, it plays key supporting roles, leading the early development of hair bundles (Tona and Wu, 2020; Kindt et al., 2012), and interacting with the overlying structures that relay head motions to vestibular hair bundles (Spoon et al., 2011; Stooke-Vaughan et al., 2012).

Cilia come in motile and immotile forms. Ciliary motility can be either the whip-like action of flagella propelling solitary cells, such as protists and sperm cells, through liquids, or it can be the collective oar-like beating of shorter cilia on epithelial cells, moving liquids along the surfaces of airways, kidney ducts, or ventricles in the brain (Ringers et al., 2020). The structures of motile and immotile cilia are quite similar, but the latter lack the molecular machinery to power movement.

The kinocilium of hair cells is a transformed primary cilium. Well before the birth of the animal, nascent hair cells look like nonspecific epithelial cells, featuring an immotile primary cilium that protrudes from the center of the top surface and is surrounded by small microvilli. As the maturing cilium glides laterally toward the cell’s edge, the microvilli also change, forming rows that elongate behind it (Tona and Wu, 2020), and acquiring the proteins that detect bundle motion (Géléoc and Holt, 2003). Although ‘kinocilium’ literally means ‘motile cilium’, sightings of its motility have been few and far between, and the consensus became that kinocilia are not normally motile.

Now, in eLife, researchers at Creighton University and the National Institutes of Health challenge this consensus with new results from mature mouse hair cells (Xu et al., 2026). Amirrasoul Tavakoli, Bechara Kachar, David He and colleagues – including Zhenhang Xu as joint first author with Tavakoli – compare two types of vestibular hair cell (types I and II), and two types of auditory hair cell (inner and outer; Figure 1A). These four classes of hair cell share many features, but they also have strikingly different functions and morphologies. Xu et al. saw differences in gene expression with the potential to contribute to known class-specific attributes. They were particularly struck, however, by specific differences related to kinocilia. Compared to the auditory hair cells, the vestibular hair cells were enriched for genes that have been associated with primary cilia and other genes associated with ciliary motility. These differences did not entirely fit with expectations.

It seems reasonable that expression of ciliary genes in mature mammalian vestibular hair cells, which retain their kinocilia, exceeds that in mature mammalian auditory hair cells, which have lost their kinocilia. However, using published datasets for immature mouse hair cells (Burns et al., 2015; McInturff et al., 2018), Xu et al. observed some vestibular enrichment for the expression of some ciliary motility genes, even though the immature auditory hair cells still have kinocilia. Together, gene expression data for ciliary genes suggest that in mammals, primary cilia and auditory kinocilia are immotile, while vestibular kinocilia have long-lasting capacity for motility.

To directly test for motility in mature vestibular hair bundles, Xu et al. turned to hair bundles from bullfrogs, which are better suited than mouse hair bundles for single-bundle imaging. They saw examples of spontaneous flagella-like motion in single kinocilia (Figure 1B). Further, electron microscopy showing the microtubular core of bullfrog kinocilia revealed stretches with structural features typical of primary immotile cilia interleaved with stretches typical of motile cilia. This suggests that the elevated gene expression in vestibular hair cells for both motile and immotile ciliary genes might reflect heterogeneity at the level of single kinocilia.

The new expression data are the first to suggest a capacity for kinociliary motility in mature mammalian hair cells, and to show a differential capacity that depends on where the hair cells come from and, by extension, what they do within the ear. How might kinociliary motility affect the detection of head motions by vestibular hair cells? In normal circumstances, it could cause problems because hair cell transduction processes cannot disentangle head motion and independent kinociliary motion.

There might, however, be abnormal situations where independent kinociliary motion would help, such as during the repair or regeneration of hair bundles. Hair cells have internal mechanisms to repair hair bundles damaged by overstimulation, and mouse vestibular organs can even generate new hair cells (Golub et al., 2012; Bucks et al., 2017). During such growth and repair processes, hair bundles are cut off from the overlying structures that normally communicate head motions to them. The interruption of normal transduction will affect calcium-mediated signaling within the hair cell. Flagellar motion of cilia is sensitive to intracellular calcium levels, providing a possible link between hair bundle repair or regeneration and kinociliary motility, which in turn may promote the return of mature bundle structure and function.

The observations and insights of Xu et al. should motivate closer characterization of the potential for functional kinociliary motility in the vertebrate inner ear. Why is it so rarely seen in vitro? How is it triggered and regulated? And what are its roles in the vestibular system we rely on to steady our balance, vision, and orientation as we move through the world?
